# Organization and expression analysis of 5S and 45S ribosomal DNA clusters in autotetraploid *Carassius auratus*

**DOI:** 10.1186/s12862-021-01918-2

**Published:** 2021-11-05

**Authors:** Chun Zhao, Yuxin Zhang, Huan Qin, Chongqing Wang, Xu Huang, Li Yang, Tingting Yu, Xidan Xu, Xiang Luo, Qinbo Qin, Shaojun Liu

**Affiliations:** State Key Laboratory of Developmental Biology of Freshwater Fish, Engineering Research Center of Polyploid Fish Reproduction and Breeding of the State Education Ministry, College of Life Sciences, Hunan Normal University, Changsha, 410081 Hunan People’s Republic of China

**Keywords:** Autotetraploid, Ribosomal DNA, Methylation, Polyploidy

## Abstract

**Background:**

Autotetraploid *Carassius auratus* (4*n* = 200, RRRR) (abbreviated as 4*n*RR) is derived from whole genome duplication of *Carassius auratus* red var. (2*n* = 100, RR) (abbreviated as RCC). Ribosome DNA (rDNA) is often used to study molecular evolution of repeated sequences because it has high copy number and special conserved coding regions in genomes. In this study, we analysed the sequences (5S, ITS1-5.8S-ITS2 region), structure, methylation level (NTS and IGS), and expression level (5S and 18S) of 5S and 45S ribosomal RNA (rRNA) genes in 4*n*RR and RCC in order to elucidate the effects of autotetraploidization on rDNA in fish.

**Results:**

Results showed that there was high sequence similarity of 5S, 5.8S and ITS1 region between 4*n*RR and RCC. This study also identified two different types of ITS2 region in 4*n*RR and predicted the secondary structure of ITS2. It turns out that both secondary structures are functional. Compared with RCC, there was no significant difference in NTS (5S rRNA) methylation level, but the expression level of 5S rRNA was lower in 4*n*RR, indicating that methylation had little effect on the expression level in 4*n*RR. IGS (45S rRNA) was hypermethylated in 4*n*RR compared to RCC, but the expression of 18S rRNA gene was no significantly different from that in RCC, indicating that methylation regulation affected gene expression in 4nRR.

**Conclusion:**

The above studies initially revealed the effects of autotetraploidization on the structure and function of 5S and 45S rRNA in *Carassius auratus*, and provided a theoretical support for the systematic study of the evolution pattern and characteristics of rDNA in vertebrates.

## Background

Polyploidy studies have reported about different aspects of life such as genome duplication, gene expression, and subsequent evolution [1, 2]. Polyploids can be classified into autopolyploids and allopolyploids. The former presents two or more homologous chromosomes in a homopolyploid which may contribute to the formation of polyvalent bodies during meiosis, whereas the latter predominantly forms bivalent pairings [3, 4]. It is worth noting that most polyploidy associated studies mainly focus on plants and less on animals. In our previous studies, we developed allotetraploid hybrids (4*n* = 148, RRBB) (abbreviated as 4*n*RB) from the first generation of *Carassius auratus* red var. (2*n* = 100, RCC) (♀) × *Megalobrama amblycephala* (2*n* = 48, BSB) (♂) hybrids [5]. In subsequent studies, abnormal chromosomal behavior during meiosis in allotetraploid hybrids (4*n*RB) led to the formation of autotetraploid sperms and autodiploid eggs, which eventually formed autotetraploid *Carassius auratus* (4*n*RR) [6, 7]. Previous researches mainly focused on allopolyploids, with only few autopolyploid studies. As the most prone to hybridization, the genomes of fish have been comprehensively studied, and thus they can be used to better understand the evolution of vertebrate cell genomes [8].

Ribosomal DNA (rDNA) is commonly used to study the molecular evolution of multigene families. In eukaryotes, rDNA genes are mainly divided into two categories: 5S rDNA and 45S rDNA repeats. rDNA encodes rRNA that represents a highly conserved gene product in all cells [9, 10]. Because of the predominance of its structure, rDNA often serves as a good resource for studying evolutionary events [4, 11]. In animals, 45S rDNA contains 18S, 5.8S, 28S, and spacers (IGS, ITS1 and ITS2), while the 5S rDNA gene is a unit consisting of a gene transcription region (120 bp) and a non-transcribed spacer (NTS). Previous studies of fish have reported that different types of 5S rDNA were due to differences in NTS (NTS I, NTS II, and NTS III) [4, 10, 12]. IGS is a transcriptional regulatory sequence of rDNA which modulates cellular processes [13, 14]. Previous analyses of rDNA repeats have mostly been carried out in invertebrates and plants. Therefore, information on 5S and 45S rDNA in vertebrates is scarce. One study reported that rRNA molecules must fold into secondary structures in order to function properly in ribosomes [15]. ITS2 provides useful biological information at a higher taxonomic level, even in all eukaryotes, because it has a conserved secondary structure [16]. Many gene promoter regions are rich in CpG, commonly known as CpG islands. Studies have shown that cytosine methylation in CpG dinucleotide guanosine 5' plays an important role in gene expression regulation [17, 18]. In this study, we analyzed the sequence, structure, methylation, and expression in 5S and 45S rRNA clusters between autotetraploid *Carassius auratus* (4*n*RR) and its parental species (RCC). From an evolutionary perspective, comparing the arrangement of the 5S and 45S rRNA in 4nRR and RCC makes sense because of their similar genomic compositions. Our results initially revealed the effects of autotetraploidization on 5S rRNA and 45S rRNA of *Carassius auratus*, and provided theoretical support for the systematic study of the evolution patterns and characteristics of rDNA in vertebrates.

## Results

### Expression sequence analysis of 5S rRNA coding region and ITS1-5.8S-ITS2 sequence

**Fig. 1:**
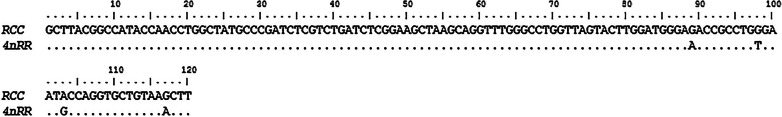
Expression sequences of 5S rRNA coding regions from RCC and 4nRR

A total of 40 copies of the gene sequences were analyzed from 4nRR and RCC. Amplification of the 5S rRNA coding region in 4*n*RR and RCC produced a 120 bp band. BLASTn alignment of the sequences in 4*n*RR detected two types: one was identical to RCC (not shown in figure), and the other had high sequence identity (average similarity of 97.5%) with corresponding sequences from RCC, although there was a few base substitution changes (Fig. 1). Therefore, our preliminary analysis showed that the 5S rRNA coding region of 4*n*RR had high similarity with the corresponding parental species sequences (GenBank Accession Nos. MZ041022 and MZ041023).

**Fig. 2:**
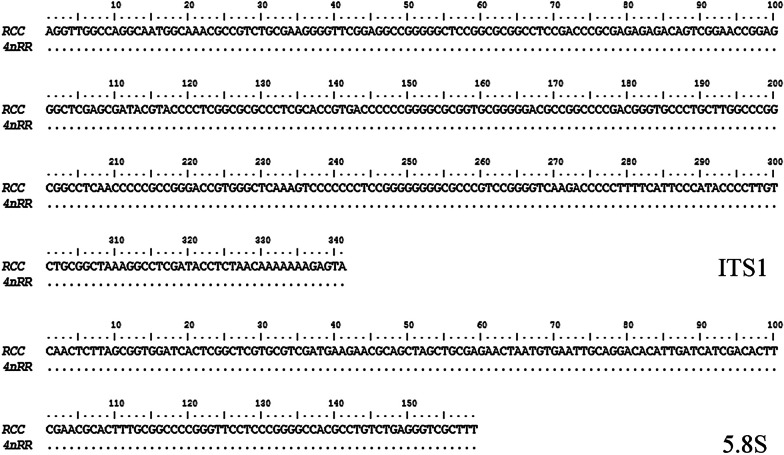
Expression sequences of ITS1 and 5.8S from RCC and 4nRR

**Fig. 3:**
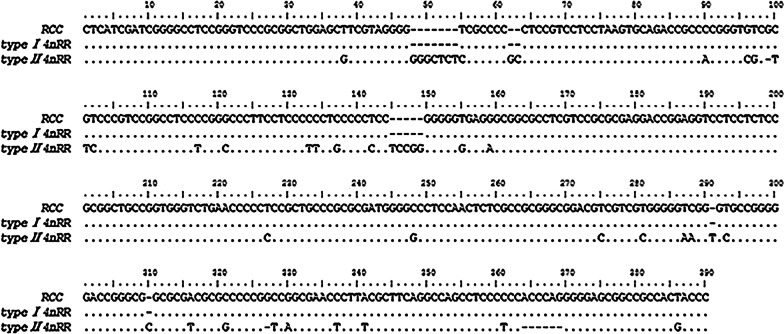
Expression sequences of ITS2 from RCC and 4nRR

It is well known that two specific sequences (called internal transcription spacers) separate the mature rRNA sequences: ITS1 (between 18S rRNA and 5.8S rRNA) and ITS2 (between 5.8S and 28S rRNA). We cloned and sequenced PCR products in order to compare the internal transcription region (ITS1-5.8S-ITS2) of 4*n*RR and RCC. For better comparison, the ITS region was divided into ITS1, 5.8S and ITS2 regions. BLASTn sequence alignments showed that the ITS1 and 5.8S rRNA of 4*n*RR had 100% similarity (Fig. 2) to RCC (ITS1: GenBank Accession Nos. MZ041015 and MZ041016; 5.8S: GenBank Accession Nos. MZ041020 and MZ041021). Nevertheless, we found two different types of ITS2 in 4*n*RR: type I (inherited from the parental species (RCC)) and type II (a newly formed type which was only expressed in tetraploid species) (Fig. 3) (GenBank Accession Nos. MZ041017-MZ041019). Figure 3 showed intraspecific variation of these sequences. Results indicated that type II ITS2 had obvious insertion, deletion and base substitution. These findings suggested that the ITS1 sequence was more conserved than ITS2.

### Prediction of ITS2 secondary structure

**Fig. 4:**
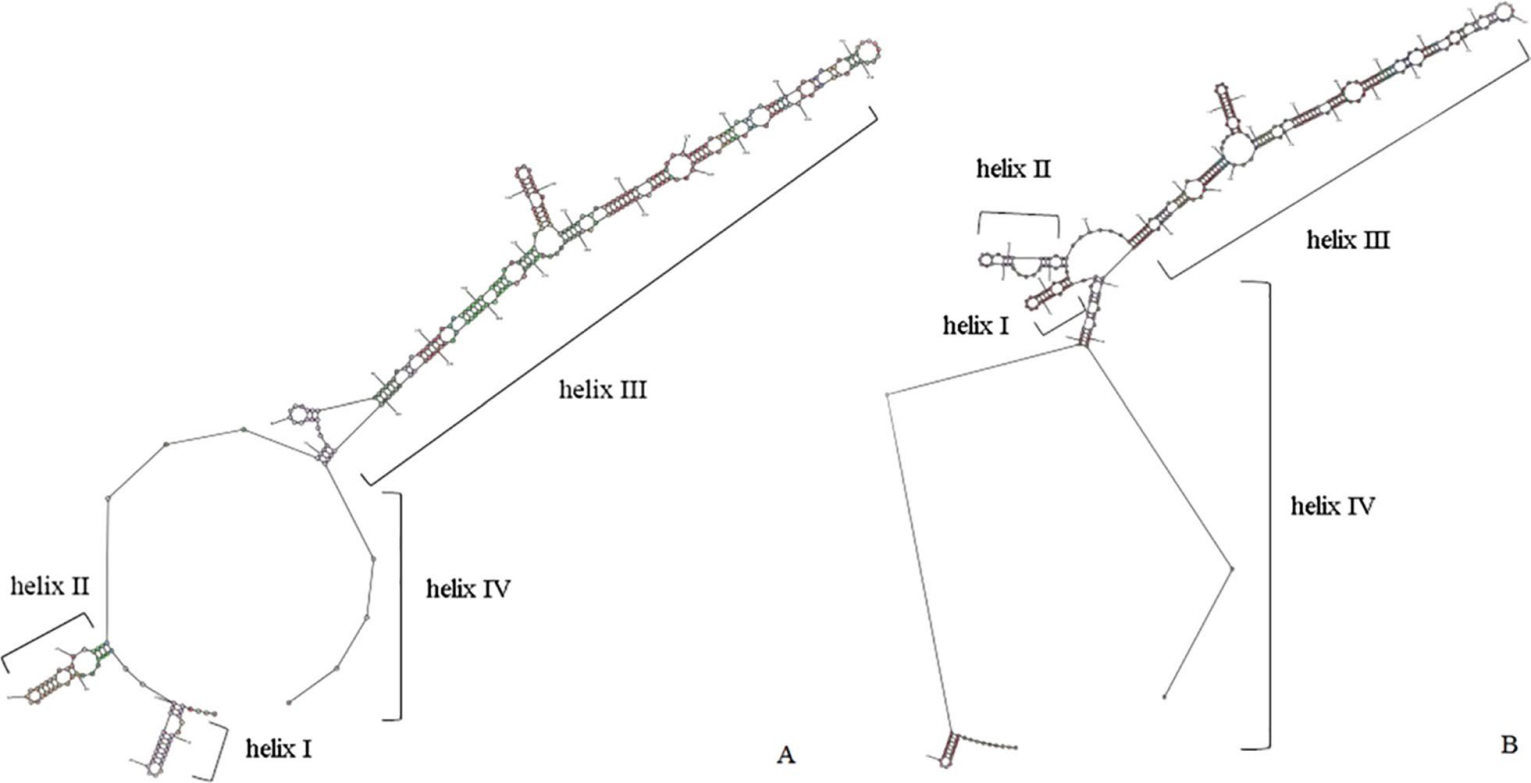
ITS2 RNA transcript secondary structures predictions in 4nRR. **A** is the secondary structure in RCC and type I. **B** is the secondary structure of type II expressed in 4nRR

ITS2 usually has four helices, but not all eukaryotes have the same number of helices. Studies have shown that only helix II and helix III are recognizable and essentially common in all organisms [16]. In this study, we predicted the secondary structure of ITS2 according to the two different sequences of type I and type II (Fig. 4). It turned out that both secondary structures were functional. The results showed that helix II (pyrimidine-pyrimidine) and helix III had high conservation in type I and type II of ITS2, especially the 5’ side of helix III (CCGGTGG).

### Expression analysis of 5S and 18S rRNA

**Fig. 5:**
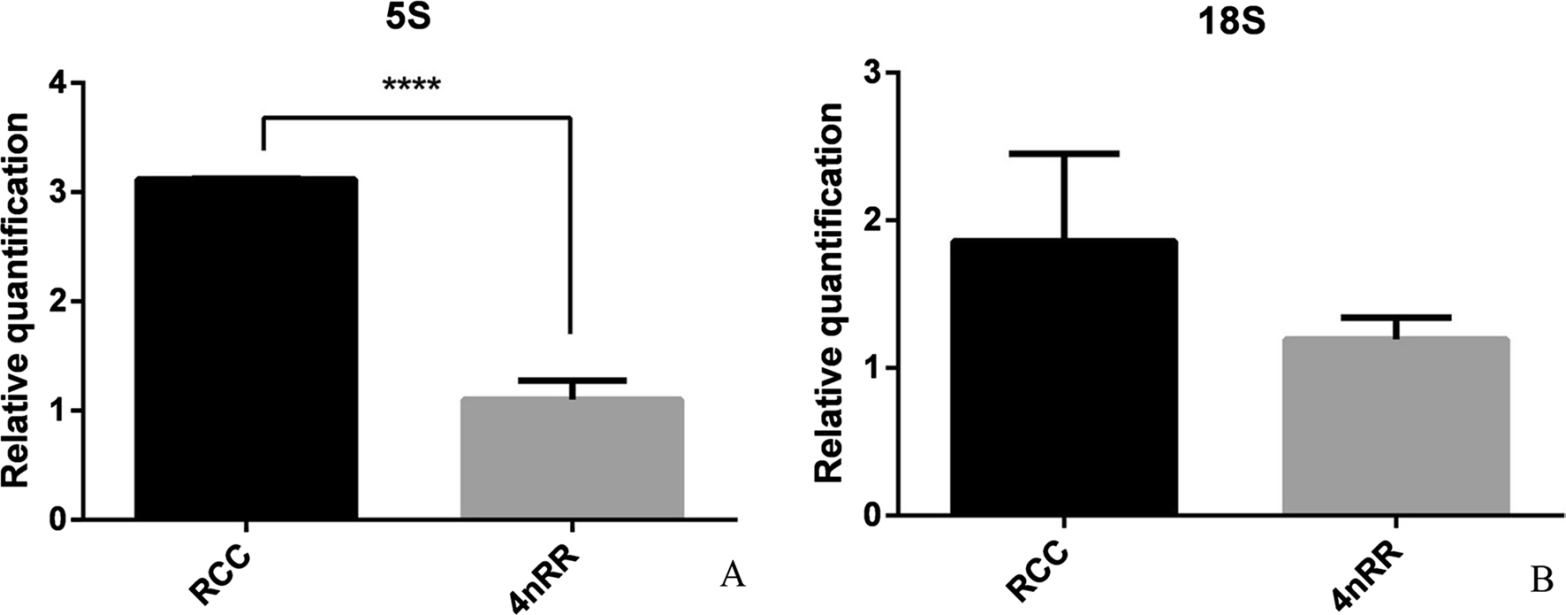
Relative expression of the 5S and 18S genes in the livers of RCC and 4nRR during the breeding season. **A** is the relative expression levels of 5S in the liver. **B** is the relative expression levels of 18S in the liver

We compared the expression of 5S and 18S rRNA genes in 4*n*RR using quantitative real-time PCR with RCC acting as the control group (Fig. 4). Results showed that the amount of 5S rRNA transcriptional products in the liver tissues of 4*n*RR was significantly lower than that of RCC group (Fig. 5A; *P* < 0.05). However, there was no significant difference in the expression of 18S rRNA gene between RCC and 4*n*RR (Fig. 5B; *P* > 0.05). These results suggested that the effects of polyploidy on the expression levels of 5S and 18S rRNA genes were not consistent.

### Methylation-specific PCR of NTS (5S rRNA) and IGS (45S rRNA)

**Fig. 6:**
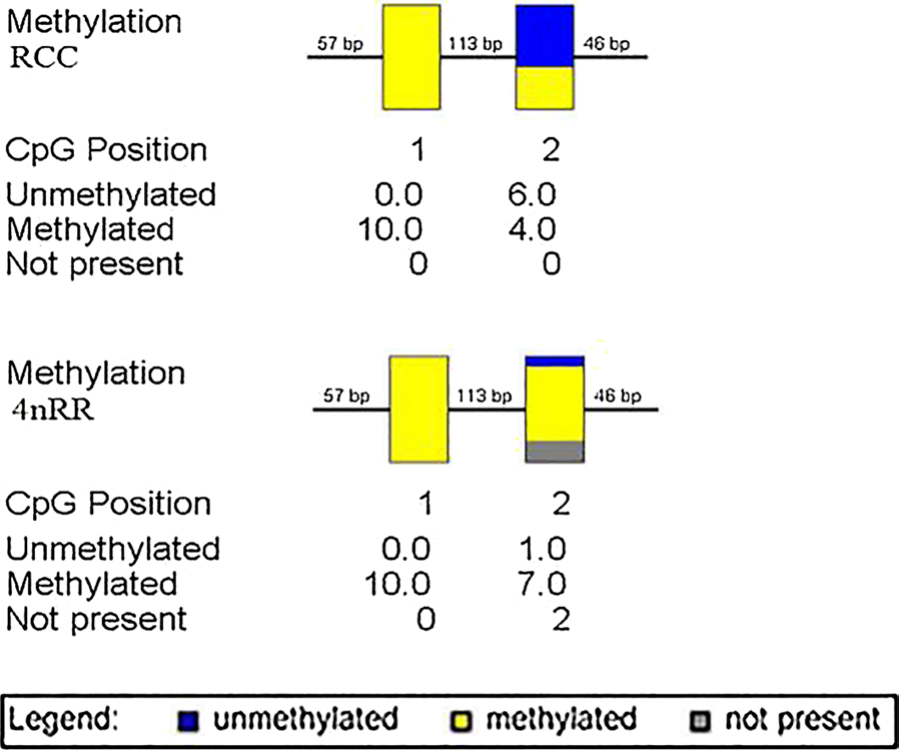
Sequencing results of methylation extent of NTS II of 5S rDNA, wherein yellow represents methylation and blue represents no methylation

**Fig. 7:**
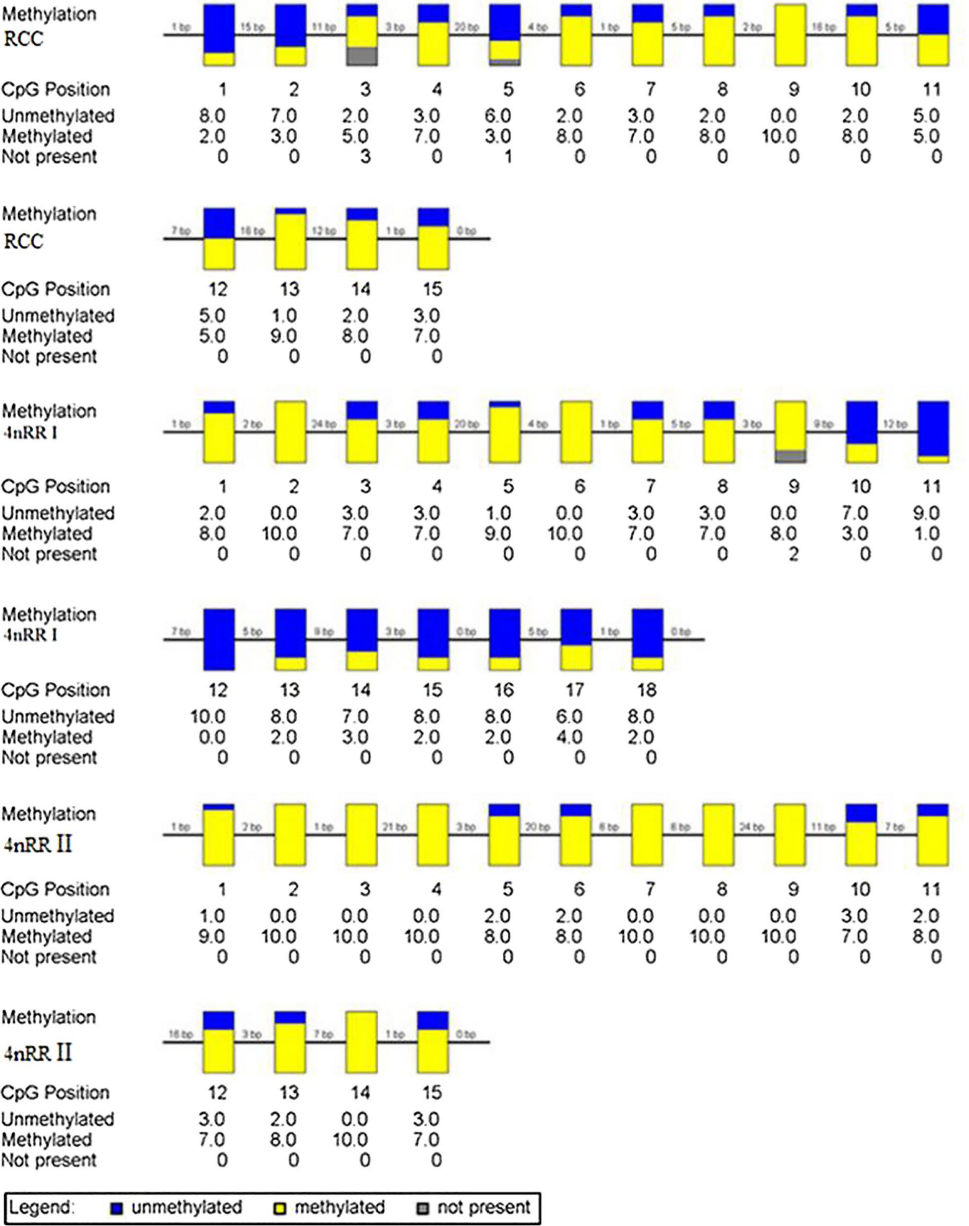
Sequencing results of methylation extent of IGS of 45S rDNA, wherein yellow represents methylation and blue represents no methylation

The results only showed differences of methylation level in NTS II because the 5S arrays of NTS I and NTS III had the same levels of methylation in RCC and 4*n*RR (Fig. 6) (GenBank Accession Nos. MZ041027- MZ041032). However, there was no significant methylation difference of NTS II between RCC and 4*n*RR (85% and 92.5%, respectively) (*P* > 0.05). Figure 7 showed analysis of the IGS methylation status of 45S rRNA in liver tissues (GenBank Accession Nos. MZ041024-MZ041026). Our results indicated that there were two different types of IGS (4*n*RR I and 4*n*RR II) in 4*n*RR. Furthermore, 4*n*RR I had a similar methylation level with RCC (*P* > 0.05), while 4*n*RR II had a higher methylation level than RCC (*P* < 0.05). In general, the IGS methylation status of 4*n*RR was hypermethylated and the degree of IGS methylation was negatively correlated with the relative expression of genes.

## Discussion

5S and 45S rDNA genes play a critical role in ribosome folding and functionality [9]. Studies have shown that the ITS region is a useful genetic marker for the analysis of intraspecific variation [19–21]. Our results indicated that the coding region of 5S rRNA gene, 5.8S rRNA gene and ITS1 region sequences were almost conserved in 4*n*RR. A previous study reported that the 5S rRNA gene (transcribed by RNA polymerase III) contained an internal control region (ICR) that acted as the promoter for the gene [22]. Generally, variation in 5S rDNA occurs in the NTS region, but the coding region remains unchanged [23, 24]. It has been reported that the ITS region (45S rDNA) participates in proper processing of ribosomal RNA sequences and forming mature functional rRNA subunits [25]. Thus, based on these results, autotetraploidization has no significant effect on the organization of 5.8S rRNA and ITS1 region. The sequences and structures are consistent, so 5.8S rRNA and ITS1 perform the same function in RCC and 4*n*RR. With regard to the ITS2 region, a comparison between RCC and 4*n*RR indicated that there were two types of ITS2 region in 4*n*RR. These mutations can be attributed to the weak selection pressure on any single copy of the gene, thereby allowing a degree of variation in the gene region [26, 27]. In addition, hybridization is accompanied by genome changes in order to overcome threats to its survival [28].

The ITS2 secondary structures presented in this study is consistent with other ITS2 structure predictions. ITS2 usually has four helices, with helix II and helix III being recognizable in almost all organisms. Helix II is very short, does not have any branches, and has a pyrimidine–pyrimidine mismatch. On the other hand, helix III is usually longer than helix II and often has branches. Previous studies reported that the largest absolute sequence conserved region in the entire ITS2 was located on the 5’ side (YCGGTGGR) of helix III close to the tip [16, 29, 30]. Moreover, these conservative characteristics are preserved in type I and type II helices. The ITS2 conserved structural motifs are necessary for all aspects of ribosomal processing [31]. The helix I is highly similar in both types. Traditionally, helix IV is the most variable region in ITS2, thus, it is normal for the two types of 4*n*RR to be different, both secondary structures are functional [8, 32]. These differences may also reflect differences in the formation of mature functional ribosomes because there are many steps involved in the production of a mature rRNA gene [25].

Newly formed polyploids undergo extensive genomic changes after genome combination and replication [33]. Polyploidy significantly affects genome formation and other genetic aspects such as gene expression. We found that there were no significant differences in expression of the 18S rRNA gene between 4*n*RR and RCC. However, the 5S rRNA gene showed significant differences. Moreover, all the genes were doubled in autotetraploid fish compared to RCC. Theoretically, if each gene was normally expressed, the total gene volume would be much higher than that of the diploid parent. This supports the findings of a previous study which reported that the origin of polyploid lineages are not consistent at the ploidy level of gene expression, with regard to increase or decrease [34]. Previous studies have shown that the genomic DNA loci of autotetraploids differ from those of diploids [35]. However, these results did not explain whether the gene expression differences were caused by genomic DNA site changes or epigenetic silencing. For example, changes in DNA methylation, a common epigenetic phenomenon, can also regulate gene expression.

To verify whether the differences between RCC and 4*n*RR were dependent on the methylation status, we analyzed the NTS methylation patterns of the different 5S rRNA arrays of RCC and 4*n*RR using the genomic sequencing technique. Previous studies have associated cytosine methylation with the non-expression of a gene [36, 37]. The 5S rRNA clusters of NTS I and NTS III in RCC and 4*n*RR were all methylated and they showed no difference in methylation status. Furthermore, although the methylation levels of NTS II varied, there was no significant difference. In summary, the methylation level in all 5S rRNA sequences was similar. These results indicated that methylation may not affect the binding of transcription factors to 5S rDNA, nor did it regulate transcription of the 5S rRNA gene. Thus, it may have no significant effect on expression of 5S rRNA gene. IGS, as a variable part of 45S rDNA, usually contains enough variation to allow examination of genetic relationships between closely related species [14, 38, 39]. In the study, there were two types of IGS in 4*n*RR: type I was hypomethylated, while type II was typehypomethylated. The results ensured that the methylation level was consistent in 4*n*RR. Among them, there was no significant difference between type I and RCC, while type II showed significant differences and a higher methylation degree than RCC. The results showed that IGS methylation was negatively correlated with relative gene expression, and methylation inhibited the expression level to some extent. The emergence of two types of IGS can be attributed to the fact that the establishment of nucleolar dominance requires several generations of selection and screening during the homologous polyploidization process. It is possible that the inhibitory mechanism that controls nuclear dominance in hybrids also control the number of active 45S rRNA gene in pure breeds and may reflect the dose compensation mechanism [11, 40, 41]. However, regulation of the active 5S rRNA gene may be different. Our quantitative real-time PCR results indicated that the expression of 5S rRNA gene was low in all 4*n*RR individuals, while the expression level of 18S rRNA gene showed no significant difference between RCC and 4*n*RR. In our previous studies, we observed loss of chromosomal sites in the generation of 4*n*RR [35]. As regulatory regions, NTS and IGS regulate gene expression during the late stage according to methylation. This phenomenon might explain why the number of chromosomes in 4*n*RR increased but there was no positive increase in the expression level. In addition, 45S and 5S rRNA could not make many differences in number because they need to form the large and small subunits of the ribosome. Otherwise, the subunits would not be paired quantitatively.

rDNA is an important component of nuclear structure and mechanisms that maintain genomic integrity [42–44]. 5S rRNA and partitial ITS sequences are still conserved during the autotetraploidization, the study has revealed that the basic unity of rDNA sequences in 4*n*RR and RCC. One study reported that the high transcriptional and recombination rates of rDNA contributed to the diversity of the genome and formation of reproductive barriers [8]. Moreover, the repetitive nature of rDNA and other duplicated genes leads to a high degree of evolutionary dynamics [45, 46]. Therefore, this tetraploid lineage can be an attractive model for elucidating genomic changes associated with autotetraploidization. Our results will expand the understanding of homologous polyploidy effects on ribosomal DNA and have important significances for the evolutionary study of polyploid *Carassius auratus*. In addition, the information on the sequences and structures of 4*n*RR (5S and 45S rRNA) provides a reference for further studies on the evolution of rDNA in fish and other vertebrates.

## Conclusion

By comparing and analyzing the sequences, structures, expression levels and methylation levels of ribosomal RNA genes (5S rRNA, 45S rRNA) in autotetraploid *Carassius auratus* (4*n*RR), we found that 5S rRNA, 5.8S rRNA and ITS1 were highly conserved, but autotetraploidization promoted the structural differentiation of ITS2 in 4*n*RR. The expression levels and methylation results showed that the methylation of the 5S rRNA regulation region did not regulate the expression of the gene, but the 45S rRNA regulation region affected the expression of 18S rRNA gene in 4*n*RR to some extent. Polyploidization is one of the main driving forces of biological evolution. The datas from this study provide some references for studying the evolution of ribosomal DNA in autopolyploid species.

## Materials and methods

### Materials

Experimental fishes were provided by the Engineering Center of Polyploid Fish Breeding of the National Education Ministry, Hunan Normal University.

### Expression sequence and expression analysis of 5S rDNA

Our analyses involved sequencing of 30 clones for each accession. Genomic DNA was isolated from blood of all samples using genomic DNA extraction kit (Takara). PCR was then performed with a specific primer complementary to the 5S rRNA conserved coding region. The primers were synthesized according to the method described by Qin et al. [47]. Primer sequences were: GCTATGCCCGATCTCGTCTGA (5′-3′) and CAGGTTGGTATGGCCGTAAGC (5′-3′). The PCR program included 30 cycles of denaturation at 94 °C for 1 min, annealing at 59 °C for 35 s, and elongation at 72 °C for 35 s. Final extension was performed at 72 °C for 15 min. Moreover, RNA was extracted from liver tissues using Trizol reagent in accordance with the manufacturer’s instructions (Invitrogen, San Diego, CA). Next, the RNA was reverse transcribed to cDNA using the PrimeScript™ RT reagent kit (Perfect Real Time, Takara) with a gDNA eraser. The 5S rRNA gene-specific primer (5′-CAGGTTGGTATGGCCGTAAG-3′) was then used to amplify the first-strand cDNA.

Amplification products were analyzed using 1–1.2% agarose gel electrophoresis stained with ethidium bromide. The PCR products were then cloned, followed by selection of clones with inserts of the predicted length (203 bp) for sequencing. Next, Bioedit and ClustalW software was used to analyze the sequence homology and variation of the amplified fragments of 4*n*RR and RCC. To determine gene expression differences, quantitative real-time PCR (Prism 7500 sequence detection system, ABI) was used to analyze the expression level of the target genes. Relative gene expression was normalized to the expression of β-actin gene, an endogenous control.

### Expression sequence (ITS1-5.8S-ITS2) and expression (18S) analysis of 45S rDNA

For amplification of ITS1-5.8S-ITS2, the following primers were used: 5′-AGTCGTAACAAGGTTTCCGTAGGTG-3′ and 3′-TTATGGCCGTGCTCTGGCTAT-5′ [11]. PCR was carried out using the conditions described above but with exception of the annealing temperature (57 °C). Moreover, the 18S rRNA gene-specific primer (5′-CATCTAAGGGCATCACAGAC-3′) was used to amplify the first-strand cDNA. Sequences and expression analysis were conducted according to a previously described protocol [48].

### Secondary structure of ITS2 sequences

We conducted comparative sequence analysis to elucidate the secondary structure of ITS2 sequences. More information about species relatability and intraspeciality variation was obtained by examining the functional folding patterns or secondary structures of the rRNA regions of interest [8, 21]. We determined the structure with the lowest free energy and compared the secondary structure of ITS2 cloned by 4*n*RR.

### Methylation-specific PCR

Using the common carp genome as a reference, we identified the spacer regions (NTS and IGS) of 5S and 45S rRNA genes in NCBI database. Sequences of the corresponding target NTS and IGS were retrieved from RCC genome (DDBJ/EMBL/GenBank Accession No. PRJNA289059) and 4*n*RR genome (unpublished), respectively. Genomic DNA was extracted from liver tissues using Sangon Animal Genomic DNA extraction kit (*n* = 3 fishes per treatment). Next, the extracted DNA was treated according to the methylcoded bisulfite conversion kit protocol (Thermo Fisher). Gene-specific primers for NTS (NTS I, NTS II, and NTS III) and IGS (Table 1) were designed using Primer 5.0 software. PCR products were ligated, transformed, and sequenced. Finally, sequences obtained from methylation results were retrieved using BiQ analyzer.

**Table 1:** Primers used in methyl-specific PCR

Primer name	Sequence
For clonig sequence	
NTS-F	5′-GCTATGCCCGATCTCGTCTGA-3′
NTS-R	5′-CAGGTTGGTATGGCCGTAAGC-3′
IGS-F	5′-GGGTGGCGGCGTCTGATAGA-3′
IGS-R	5′-CCCAAACTTCAGGATTTGTGC-3′
For methyl-specific PCR	
NTS I-F	5′-CGGAAGTTAAGTAGGTTTGGGT-3′
NTS I-R	5′-GTAAACGAAAACTACTACAAAA-3′
NTS II-F	5′- GAATATTAGGTGTTGTAAGTT-3′
NTS II-R	5′-AACCGTAAACGAAATCTACTA-3′
NTS III-F	5′-TTGGGAATATTAGGTGTTGTAA-3′
NTS III-R	5′-TAAACGAAAACAACTACAAAAA-3′
IGS RCC-F	5′-GYGTTTGATAGAGGGTTAYGGGGTTT-3′
IGS RCC-R	5′-TAAAAACCCRTCAACCCCTCTCAAAC-3′
IGS 4nRR I-F	5′-GTTGTATTTYGGTTTTTTTGGGGGTT-3′
IGS 4nRR I-R	5′-TAATAAAAACCCGTCAACCCCTCTCA-3′
IGS 4nRR II-F	5′-TTTTGGTTTTYGGTGGTGTGGGGATT-3′
IGS 4nRR II-R	5′-TCTCAACRACRCCRAAACCCAAAAAC-3′

## Data Availability

All data generated or analysed during this study were included in this published article. The sequence for these libraries have been uploaded to the NCBI Sequence Read Archive site (http://www.ncbi.nlm.nih.gov/sra/; accession nos.): ITS1 (GenBank Accession Nos. MZ041015 and MZ041016); ITS2 (GenBank Accession Nos. MZ041017-MZ041019); 5.8S (GenBank Accession Nos. MZ041020 and MZ041021); 5S (GenBank Accession Nos. MZ041022 and MZ041023); IGS (GenBank Accession Nos. MZ041024-MZ041026); NTS (GenBank Accession Nos. MZ041027- MZ041032). All data have been stored in the NCBI database, and will postpone the release on April 24, 2022.

## Abbreviations

RCC: *Carassius auratus* Red var.
BSB: *Megalobrama amblycephala*
4*n*RB: Allotetraploid hybrids
4*n*RR: Autotetraploid *Carassius auratus*
NTS: Non-transcribed spacer
IGS: Internal transcribed spacer
rDNA: Ribosomal DNA
rRNA: Ribosomal RNA
